# Alcohol exposure decreases osteopontin expression during fracture healing and osteopontin-mediated mesenchymal stem cell migration in vitro

**DOI:** 10.1186/s13018-018-0800-7

**Published:** 2018-04-27

**Authors:** Roman M. Natoli, Henry Yu, Megan Conti-Mica Meislin, Pegah Abbasnia, Philip Roper, Aleksandra Vuchkovska, Xianghui Xiao, Stuart R. Stock, John J. Callaci

**Affiliations:** 1 Department of Orthopaedic Surgery and Rehabilitation, Stritch School of Medicine, Loyola University Chicago, 2160 South First Ave, Maywood, IL 60153 USA; 2 Present Address: Department of Orthopaedic Surgery, Indiana University School of Medicine, Indianapolis, IN USA; 3 Present Address: Department of Orthopaedic Surgery and Rehabilitation Medicine, Hand and Upper Extremity Division, The University of Chicago, Chicago, IL USA; 4 Present Address: School of Veterinary Medicine, University of Pennsylvania, Philadelphia, PA USA; 5 Present Address: Argonne National Laboratory Advanced Photon Source, Lemont, IL USA; 6 Present Address: School of Medicine, Northwestern University Feinberg, Chicago, IL USA

**Keywords:** Bone fracture, Fracture non-union, Alcohol, Osteopontin, Integrin, Mesenchymal stem cell migration

## Abstract

**Background:**

Alcohol consumption is a risk factor for impaired fracture healing, though the mechanism(s) by which this occurs are not well understood. Our laboratory has previously shown that episodic alcohol exposure of rodents negatively affects fracture callus development, callus biomechanics, and cellular signaling which regulates stem cell differentiation. Here, we examine whether alcohol alters chemokine expression and/or signaling activity in the mouse fracture callus during early fracture healing.

**Methods:**

A mouse model for alcohol-impaired tibia fracture healing was utilized. Early fracture callus was examined for alcohol-effects on tissue composition, expression of chemokines involved in MSC migration to the fracture site, and biomechanics. The effects of alcohol on MSC migration and cell adhesion receptors were examined in an in vitro system.

**Results:**

Mice exposed to alcohol showed decreased evidence of external callus formation, decreased callus-related osteopontin (OPN) expression levels, and decreased biomechanical stiffness. Alcohol exposure decreased rOPN-mediated MSC migration and integrin β1 receptor expression in vitro.

**Conclusions:**

The effects of alcohol exposure demonstrated here on fracture callus-associated OPN expression, rOPN-mediated MSC migration in vitro, and MSC integrin β1 receptor expression in vitro have not been previously reported. Understanding the effects of alcohol exposure on the early stages of fracture repair may allow timely initiation of treatment to mitigate the long-term complications of delayed healing and/or fracture non-union.

## Background

While most patients suffering a bone fracture enjoy an uncomplicated recovery, impaired fracture healing [delayed union, non-union] occurs in approximately 5–10% of patients [1], with up to 19% of patients with open tibial shaft fractures progressing to non-union. [2]. There are several factors that contribute to impaired fracture healing, one of which is excessive alcohol consumption [3–6]. Patients with non-unions have increased morbidity [7] and often require further surgical interventions, which have limited efficacy and are costly to the healthcare system. Understanding the biology of alcohol-impaired fracture healing may lead to the development of non-surgical strategies to prevent or reverse the process.

Alcohol consumption affects bone remodeling [3, 8], and rodent studies have documented the deleterious effects of chronic alcohol administration on fracture healing [9–11]. Our laboratory has demonstrated that episodic alcohol exposure negatively affects both bone remodeling and the healing of experimentally induced fractures in rodents, and appears to specifically affect cartilaginous callus formation [8, 12–23]. Cartilaginous callus formation depends on the presence and activity of mesenchymal stem cells (MSC) at the fracture site. Wezeman and colleagues [24, 25] demonstrated that alcohol exposure inhibited the in vitro osteogenic differentiation potential of primary cultured human MSC. Stem cells have the ability to migrate following injury, and work shows that MSC home to the site of a healing fracture [26–29]. While the exact role of these migrating cells in fracture healing has not been determined, two chemokines, stromal cell-derived factor-1 (SDF-1α) [30], and osteopontin (OPN) [31] induce MSC homing following injury. Reports suggest that OPN, specifically through interaction with the integrin β1 receptor, may regulate MSC migration [24, 32]. The effects of alcohol on MSC migration following fracture have not been examined nor has any work examined the effects of alcohol on OPN-related signaling activity following fracture.

Our laboratory has demonstrated that the localization of exogenously delivered MSC to the fracture site may differ between control and alcohol-exposed mice [20]. We hypothesized that one potential mechanism underlying the inhibition of cartilaginous callus formation observed in alcohol-exposed rodents could be related to a disruption of SDF-1 and/or OPN expression in fracture callus of animals exposed to alcohol. We further hypothesized that perturbations of fracture site-associated chemokine expression in alcohol-exposed animals would be associated with changes in fracture callus tissue composition and structural properties. In an effort to link alcohol exposure to MSC activity, we utilized an in vitro system to test the hypothesis that alcohol treatment attenuates the migration of primary cultured rodent MSC.

## Methods

This study investigates the effects of alcohol exposure on the early stages of fracture healing using a mouse model of tibia fracture. This study received approval in 2012 from the Loyola University Chicago, Institutional Animal Care and Use Committee (IACUC #12–057). Sixty-six wild type (C57BL/6) male mice aged 6–7 weeks were obtained from The Jackson Laboratory (Bar Harbor, ME). Mice were acclimated for 1 week in our animal care facility prior to initiation of the experiment and were randomly assigned to either the saline control or alcohol exposure treatment groups.

### Alcohol exposure

Mice received either intraperitoneal (IP) injections of 20% (*v*/*v*) ethanol/sterile isotonic saline solution made from 100% molecular grade ethanol (Sigma-Aldrich, St. Louis, MO) at a dose of 2 g/kg, or sterile isotonic saline at similar volumes. The alcohol exposure regimen was once daily IP injections given for 3 days 1 week prior to fracture, and then again, the 3 days leading up to fracture (4 days between injection cycles). Using this dosing regimen, a blood alcohol level (BAL) of ~ 200 mg/dl was achieved 1 h post injection, (at the time of fracture injury) to mimic the heavy episodic drinking patterns observed in intoxicated trauma patients [33]. Alcohol administration was continued during the post-fracture period to mimic patient post-trauma alcohol consumption patterns [16, 34].

### Fracture surgery protocol

Mouse tibia fractures were created as previously described [18]*.* Briefly, anesthesia was induced with a combination of intraperitoneal ketamine (0.75 mg/kg) and xyalzine (0.08 mg/kg). Animals were prepped for sterile surgery, given prophylactic gentamicin (5 mg/kg) and anesthetized with inhaled isoflurane. An incision was made over the left proximal tibia, skin was retracted proximally to expose the patellar tendon, and a 27 G needle was used to gain access to the tibia intramedullary canal from a lateral parapatellar position. A stainless pin (0.25 mm, Fine Science Tools, Foster City, CA) was inserted into the tibial canal to stabilize the bone. The incision was retracted distally to overlie the mid tibial diaphysis and angled bone scissors were used to create a mid-shaft transverse fracture. The pin was cut flush with the proximal tibia and the wound was sutured. Mice were given 1 cc of saline subcutaneously for resuscitation. All mice received three doses of buprenorphrine (0.05 mg/kg) subcutaneously for pain control q8 hours post-operatively. By 24 h post-operatively, mice were active and weight bearing on the injured limb.

### Specimen processing

Fractured and contralateral tibiae were harvested from mice following euthanasia at 3 or 7 days post-fracture. Fracture callus specimens harvested at 3 days post-fracture were utilized for either histology or chemokine protein expression analysis. Fragility of callus specimens at 3 days post-fracture did not allow for biomechanical testing or Micro-CT analysis at this time point, so callus specimens harvested at 7 days post-fracture were utilized for biomechanical, Micro-CT as well as chemokine analysis. Care was taken to dissect all visible soft tissue from the callus of the fractured limb. Tibiae taken for biomechanical testing were wrapped in saline soaked gauze and stored at − 20 °C. Samples for histology or micro CT testing were placed into 10% neutral buffered formalin and stored at room temperature. Samples taken for protein analysis were snap frozen in liquid nitrogen and stored at − 80 °C.

### Gross morphology and histology

**Fig. 1:**
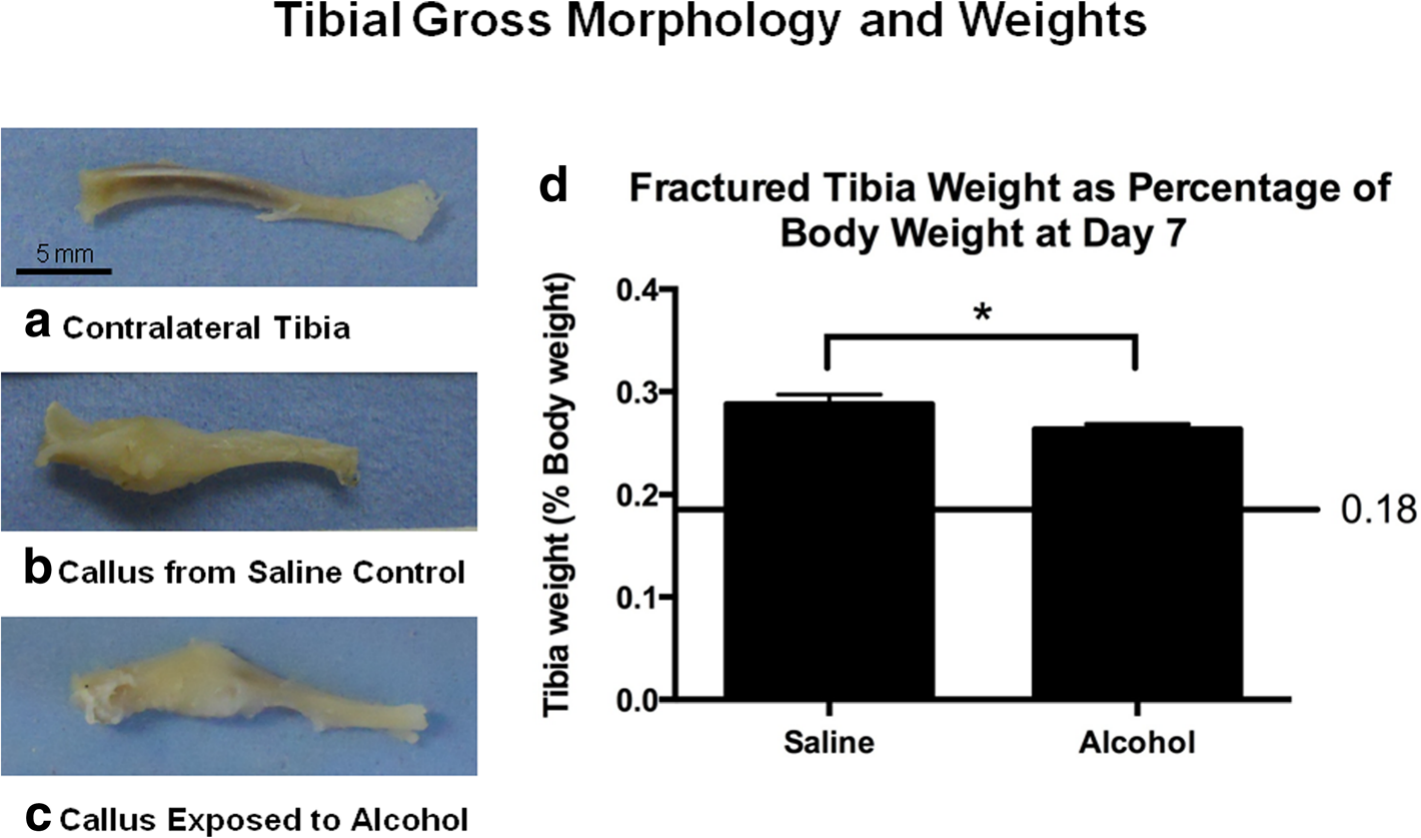
Tibia fracture morphology and weights. **a** Contralateral intact tibia from a saline control mouse. **b** Fracture callus in situ at 7 days post-fracture from a saline control mouse. **c** Fracture callus in situ at 7 days post-fracture from an alcohol-exposed mouse. The calluses from the saline control and alcohol-exposed mice were similar in size, but the alcohol-exposed callus was less robust appearing. Scale bar in **a** represents 5 mm and applies to **b** and **c** as well. **d** Tibial weight at 7 days post-fracture as a percentage of the mouse total body weight (tBW). The line represents intact contralateral limbs, which were 0.18 ± 0.01% tBW for both saline control and alcohol-exposed mice. Data are shown as mean ± SEM, *n* = 9/group. *p = 0.03 by Student’s *t* test

Photographs of gross morphology were taken of tibiae prior to biomechanical testing (Fig. 1). For histology, specimens were fixed in 10% formalin for a minimum of 7 days and then decalcified in 10% EDTA with agitation for 7 days. Sagittal sections were stained with H&E and were mounted on glass slides.

### Sample preparation and protein analysis

Samples were removed from − 80 °C and were placed on dry ice. Whole tibia, whether fractured or intact contralateral, were weighed. Fracture callus was isolated from injured tibiae using a Dremel rotary cutting tool (Dremel, Racine, WI); contralateral intact tibiae were left undisturbed. A Spex Freezer Mill (SPEX, 6770 SamplePrep, Metuchen, NJ) was then used to pulverize the specimens while frozen in 1 mL lysis buffer (from 10 mL RIPA Buffer, 1 tablet Protease Inhibitor Cocktail, 100 μL Halt Phosphatase Inhibitor). Total protein in the samples was measured using the Pierce™ BCA assay (ThermoFisher Scientific, Rockford, IL). OPN and integrin β1 protein levels were measured via western blot. 15 μg total protein per sample was resolved on a 4 to 20% SDS-PAGE gel, was transferred to a PVDF membrane, and was probed with either the Phosphoprotein 1 (SPP1 or Osteopontin 1) rabbit anti-mouse monoclonal antibody (Epitomics, Burlingame, CA) or the anti-integrin β1 rabbit polyclonal antibody (abcam, Cambridge, MA). To assess protein transfer, the membranes were stained with Coomassie blue [18] after total OPN detection (~ 33 kDa). Densitometric analyses were carried out using Image Lab software (Bio-Rad, Hercules, CA). Total OPN values were normalized to a ~ 40 kDa band on the Coomassie stained membrane. SDF-1α was assayed using the mouse CXCL12/SDF-1α Quantikine ELISA (R&D Systems, Minneapolis, MN). R&D Systems Spike and Recovery protocol for validating untested samples was used to confirm test validity (data not shown).

### Biomechanical testing

Injured and contralateral tibiae, harvested from control and alcohol-exposed mice at 7 days post-fracture, were used for 4-point bending analysis. The contralateral tibias served as the uninjured control group. Samples were thawed at room temperature and were loaded into a customized 4-point bending apparatus (long-span distance 7 mm; short-span distance 3 mm) and tested at 0.5 mm/s using a biomaterial testing machine (Model 5544; Instron Corporation, Canton, MA). Calluses were centrally positioned within the short span. Load-deflection curves were obtained, and the slope of the linear portion was taken as the callus stiffness.

### Micro-CT analysis

Specimens were placed into a tube containing 100 μL of formalin with a small wick of gauze at the base. High-resolution phase contrast synchrotron μCT was performed with the Argonne National Laboratories Advanced Photon Source beamline 2-BM. Synchrotron μCT produces images with sharper features and phase contrast improves sensitivity to different soft tissue types [35], allowing easier/more accurate segmentation of soft tissue. Pilot scans showed a 600-mm distance between the specimen and detector optimized contrast between air and soft tissue compared to other separations. Final imaging parameters were 24.3 keV, 600 mm imaging distance, × 2.5 lens, 0.12° rotation between image acquisition with 300 ms exposure, and a (2 K)^2^ CCD. Reconstructions consisted of 2.8 μm isotropic voxels. Data was reconstructed using a customized in-house program similar to ANKAphase [36] based on the Paganin single-distance phase retrieval algorithm [37]. To minimize selection bias, specific parameters were established for selecting the portion of fracture callus to be analyzed. The distal end was set where the cross-sectional area was 3.9 mm^2^. A fixed length of 2.98 mm of callus proximal to the distal end was analyzed for each sample. Between the proximal and distal ends, there were 1065 slices. We measured callus volume every 15th slice and interpolated the callus volumes between measurements. The total volume (TV) of the callus was defined as the volume of all voxels within the callus, was performed by manually outlining the border of each specimen to define the region of interest. To quantify tissue composition in the ROI, 32 B, grayscale images were established by visual segmentation. Absolute numbers for the calculated volumes did not change with slight shifts of the threshold values, and changes observed when the thresholds were varied were similar among specimens. Bone volume (BV) was defined between 0.000691 and 0.00417 on the image histogram, mineralized tissue was from 0.0000619 to 0.000691, and soft tissue was defined as the remaining tissue within the ROI less than 0.0000619 on the image histogram. These thresholds were applied to each sample, and the volumes were calculated using the BoneJ plugin [38] for ImageJ. In addition to the volume, the polar moment of inertia (*I*_pol_) was calculated using the slice geometry function in BoneJ. *I*_pol_ values were averaged over the 71 sections as previously described [39]. The polar moment of inertia measures the distribution of mass in a cross section of a material, serving as a description of the geometry of the callus and is proportional to its resistance to bending.

### In vitro MSC migration

Primary mice (C57BL/6) Mesenchymal Stem Cells (Invitrogen, Carlsbad, CA) were used for the migration assay. Cells were used for all experiments at Passage 9. MSC were added to a growth medium consisting of DMEM/F-12 medium with GlutaMAX™-I, 10% MSC-Qualified FBS, and 5 μg/mL gentamycin. Cells were then incubated at 37 °C in 5% CO_2_ on flasks seeded at 5000 cells/cm^2^ until plates were ~ 90% confluent. MSC were detached using a TrypLE solution (Life Technologies, Grand Island, NY), were washed twice with sterile PBS, and then were resuspended in medium (DMEM + 0.1% BSA) at a concentration of 30,000 cells per 0.04 mL. The in vitro cell migration assay was carried out using ChemoTx® disposable chemotaxis system 96-well plates with 8-μm pore size (NeuroProbe, Gaithersburg, MD). Upper wells were loaded with 30,000 MSC suspended in medium. Medium with recombinant murine OPN (R&D Systems) at concentrations of 1 and 5 μg/ml was added to the lower wells. Medium alone was used as the negative control. After 24 h of incubation, cells remaining on the top surface of the membrane were removed. Cells migrating to the bottom surface of the membrane were fixed using 2.5% glutaraldehyde, stained with hematoxylin, and counted under a light microscope. Each assay condition was carried out in triplicate, and the average value was reported. The assay was repeated four times with different MSC cultures. The assay conditions tested were (1) MSC cultured with 50 mM ethanol, no ethanol during migration assay (2) Ethanol present only during migration (50 mM ethanol added to the lower wells), and (3) 24 h of MSC cultured in the presence of 50 mM ethanol and ethanol added to lower assay well. 50 mM ethanol is equivalent 230 mg/dL, equivalent to the BAL of mice at the time of fracture surgery.

### MSC isolation

Mesenchymal stem cells were isolated from 6 to 7-week-old male Lewis rats using a modified protocol as described previously [40, 41]. Briefly, animals were humanely euthanized, and both tibiae and femurs were harvested. The proximal and distal ends of each bone were sheared off with bone snips. The marrow of each bone was flushed with D-MEM supplemented with 20% FBS, and the resulting marrow cell suspension was filtered through a 70-μM filter to remove any contaminating bone or cell clumps. This cell suspension was centrifuged at 450 g for 5 min; the pellet was resuspended in 5 mL of D-MEM containing 20% FBS and transferred to a T-25 cm^2^ culture flask. The culture medium was carefully replaced after 24 h of culture and then every 3–4 days as needed to retain plastic-adherent cells and to remove any contaminating non-adherent cell populations. The timing of the replacements of culture media following culture initiation to remove contaminating cell populations from the primary MSCs differs from protocols for the isolation of other related stem cell populations such as those from adult muscle tissue, in which media changes are not performed until later (5 days) when plastic adherent cells of myogenic origin are established [42]. The MSCs were sub-cultured before colonies became multilayered. After one passage for expansion, the cells were then harvested and aliquoted at 1 million cells/mL in freezing medium (DMEM supplemented with 20% FBS and 10% DMSO) and deposited in liquid nitrogen vapor phase storage.

### Integrin beta1 expression

Rat MSCs were cultured in low glucose, GlutaMAX™ D-MEM (Gibco, ThermoFisher Scientific, Rockford, IL) supplemented with 10% FBS (Gibco, ThermoFisher Scientific). Cells were grown in 75 cm^2^ culture flasks until approximately 80% confluent. Cells were then exposed to media alone or media plus 50 mM EtOH for 24 h with EtOH loss by evaporation mitigated by culturing in a sealed system with excess EtOH at the same concentration as the treatment. Integrin β1 mRNA and protein expression were measured using qRT-PCR and western blotting, respectively. For both, cells were harvested using TrypLE Express (1×, ThermoFisher) and pelleted by centrifugation. Then, either total RNA was isolated using the Qiagen RNeasy Mini Kit (Qiagen, Carol Stream, IL) or total protein was isolated using 1 mL Lysis Buffer (from 10 mL RIPA Buffer, 1 tablet Protease Inhibitor Cocktail, 100 μL Halt Phosphatase Inhibitor). RNA was quantified using a NanoDrop ND-1000 spectrophotometer and quality assessed with the Agilent 2100 Bioanalyzer (Agilent Technologies, Santa Clara, CA). RNA was used to create a cDNA library (High-Capacity cDNA Reverse Transcription Kit, ThermoFisher). cDNA libraries were subjected to quantitative real-time PCR analysis (Applied Biosystems 7500 Fast qRT-PCR). The resulting data were analyzed by the delta-delta Ct method. TaqMan Fast Advanced Master Mix, compatible, and TaqMan FAM primer probes specific for integrin beta1 and beta2 microglobulin (β2M), the endogenous control were used (ThermoFisher). Integrin β1 protein expression was assayed using western blot as previously described above in *Sample Preparation and Protein Analysis*.

### Data analyses

Data are expressed as mean ± SEM. Statistical analysis was performed using Prism v6.0a (GraphPad Software, La Jolla, CA). Student’s *t* test was used to compare saline control and alcohol-exposed groups for tibial weight, μCT tissue composition, and bending stiffness. Chemokine protein expression levels were analyzed by 2-way ANOVA using injury status (intact or fracture) and treatment (saline or alcohol) as factors with Tukey’s post-hoc testing. Cell migration data were analyzed by 1-way ANOVA using pre-defined comparisons with Holm-Sidak post hoc testing. Nine pairwise comparisons were performed to examine the effects of OPN dose and alcohol exposure (see Fig. 7 legend). Protein and mRNA levels of integrin β1 were compared with Student’s *t* test. A *p* value < 0.05 was considered significant.

## Results

### Effects of alcohol on fracture callus morphology and structure

No significant effects of alcohol treatment on mouse body weight at the time of euthanasia were noted (data not shown). Figure 1 shows representative tibia samples from an uninjured saline control mouse (Fig. 1a), a fracture-injured saline control (Fig. 1b), and a fracture-injured alcohol-exposed animal (Fig. 1c) at 7 days post-injury. Figure 1d shows the tibial weight of the fractured tibia normalized to total mouse body weight (BW). Fractured tibia from mice in the alcohol-exposed group weighed significantly less (*p* = 0.03) compared to fractured tibia from saline control animals.

**Fig. 2:**
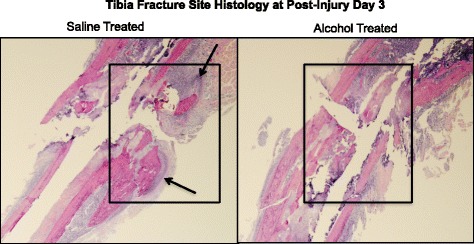
Fracture callus histology (H&E staining 10×). Histological structure of the fracture site is shown at 3 days post-fracture in saline control (**a**) and alcohol exposed (**b**). The fracture site of saline control mice shows evidence of granulation tissue accumulation (boxed area) and the presence of early cartilaginous callus formation (arrows). In contrast, the injury site of alcohol-exposed mice shows no evidence of granulation tissue accumulation or ormation or cartilage tissue. *n* = 2 per group

We have shown that episodic alcohol treatment of mice inhibits cartilaginous external fracture callus formation at post fracture days 6 and 9 [43]. Here, we examined H&E-stained sections of the fracture site in saline and alcohol-treated mice at day 3 post-injury for evidence of alcohol-related effects on early post-fracture granulation tissue accumulation (Fig. 2). The fracture site from saline control animals shows accumulation of granulation tissue (Fig. 2a, boxed areas) and early cartilage formation (arrow). In contrast, the fracture site of alcohol-exposed animals shows almost no accumulation of either granulation tissue (Fig. 2b, boxed area) or cartilage formation. Samples shown in Fig. 2 are representative for each treatment group.

### Effects of alcohol on fracture callus biomechanics

**Fig. 3:**
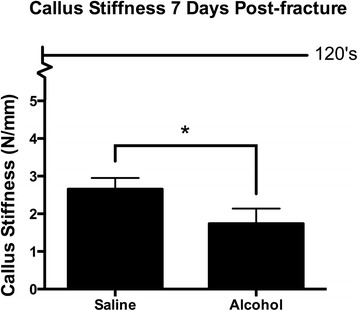
Fracture callus biomechanical analysis. Biomechanical stiffness of post-injury day 7 fracture callus from saline control and alcohol-exposed mice was assessed using a 4-point bending apparatus. The line in the graph represents stiffness of intact contralateral limbs, which were 122 ± 12 and 127 ± 13 N/mm for saline control and alcohol-exposed mice, respectively. Calluses from the alcohol-exposed mice were significantly less stiff than the saline controls. Data are shown as mean ± SEM, *n* = 9/group. *p = 0.04 by Student’s *t* test

Fractured tibia specimens were tested at 7-days post-injury for biomechanical maximum load to failure and bending stiffness using four-point bending. A large plastic deformation of the fractured tibia specimens was observed while testing, causing the specimen to wedge in the 4-point testing apparatus, making load to failure measurements unreliable at this time point (data not shown). Callus stiffness was measurable at 7 days post-injury by 4-point bending and was significantly decreased in calluses from alcohol-exposed mice compared to saline controls (Fig. 3).

### Effects of alcohol on fracture callus microstructure

**Fig. 4:**
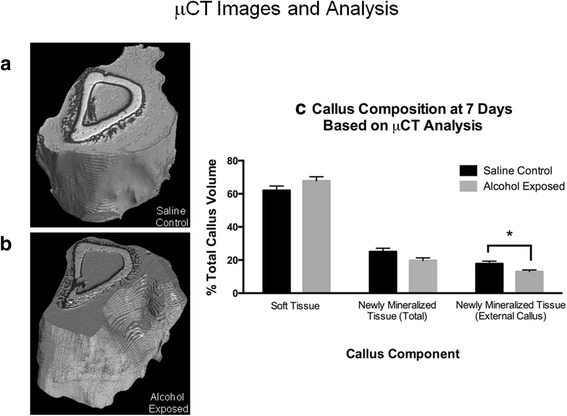
Micro-CT analysis of fracture callus at day 7 post-fracture. Representative 3D reconstructions of **a** saline control and **b** alcohol-exposed fracture calluses. The white area is mature, pre-existing bone; black area is mineralized tissue formed since fracture; gray area is soft tissue (based on segmentation as described in the “Methods” section). There is more newly mineralized tissue seen in the saline control callus than in callus from alcohol-treated mice. **c** Quantification of soft tissue, total newly mineralized tissue, and newly mineralized tissue in the external callus as a percent of the total callus volume (%TV). There is significantly less newly mineralized tissue in the external callus from alcohol-exposed mice compared to saline controls. Data are shown as mean ± SEM, *n* = 5/group. *p = 0.03 by Student’s *t* test

Fracture callus specimens from the saline control and alcohol-exposed groups were imaged with phase contrast synchrotron μCT at 7 days post-injury to determine total callus volume (TV) and the percent of callus composed of soft tissue and mineralized tissue. Figure 4a, b shows representative calluses from the saline control and alcohol-exposed groups, respectively. Within the callus, the white tissue is mature, pre-existing bone, while the black tissue is mineralized tissue formed since fracture. Gray tissue is soft tissue (based on segmentation as described in the “Methods” section). Total callus volume was not significantly different between the experimental groups, measuring 19.83 ± 0.85 and 21.29 ± 1.29 mm^3^ for the saline control and alcohol-exposed groups, respectively (data not shown). The percent soft tissue volume of the callus was not significantly different between the experimental groups, (Fig. 4c, left most bars). Total newly mineralized tissue formed since fracture in the callus (callus tissue within the medullary canal and external to the bone shaft) trended toward a significant difference (*p* = 0.08) for the saline control and alcohol exposed, respectively (Fig. 4c, middle bars). When selecting only the external compartment of the callus, there was a significant difference (*p* = 0.03) seen in newly mineralized tissue (Fig. 4c, right bars). The total volume (in percent) of newly mineralized tissue in the saline control callus was 17.8 ± 1.5, and 13.0 ± 1.0 for the alcohol-exposed group, a 27% decrease. The average polar moment of inertia (*I*_pol_) for the calluses was not significantly different between groups.

### Effects of alcohol on fracture callus OPN and SDF-1α protein levels

**Fig. 5:**
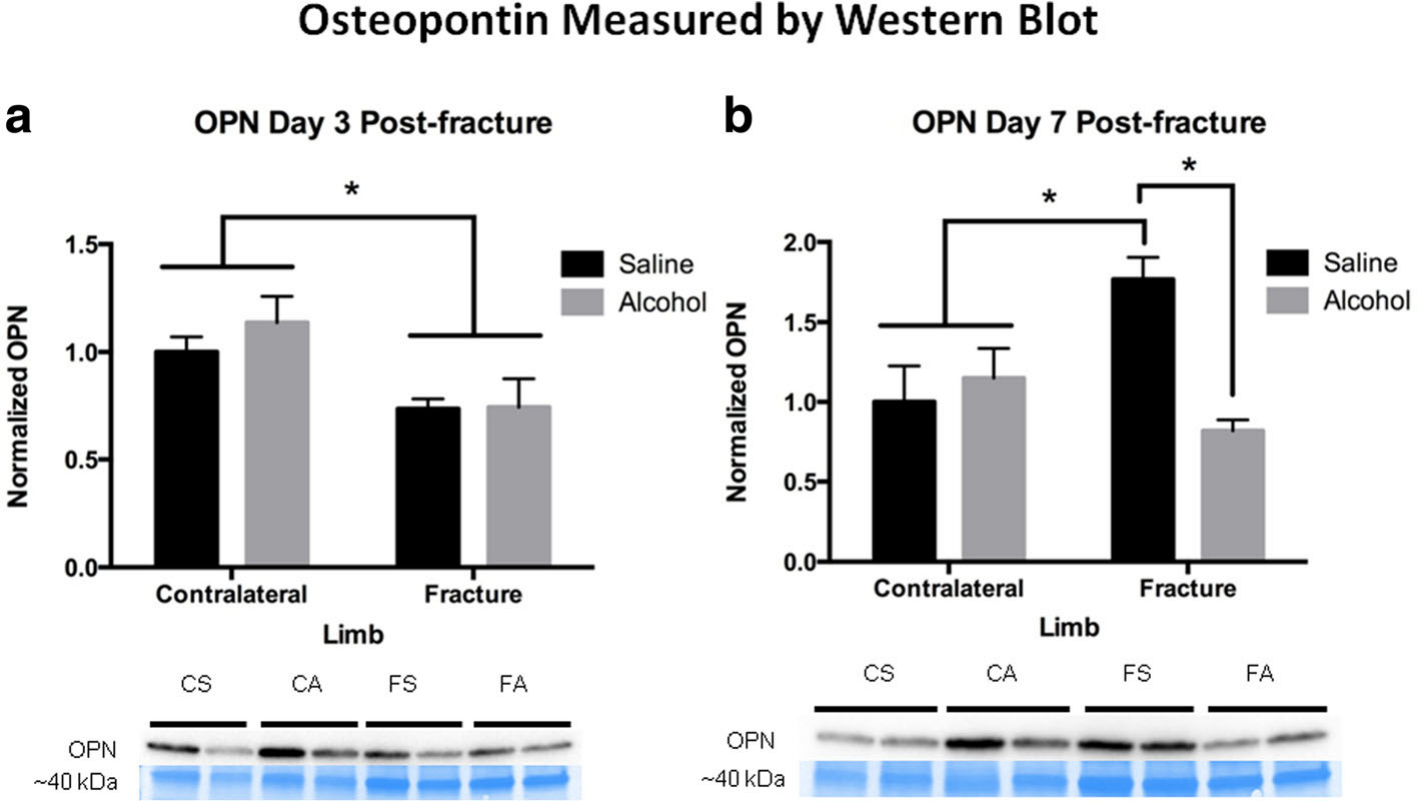
Osteopontin-1 protein levels in fracture callus at **a** 3 and **b** 7 days post-fracture. Bar graphs show fracture callus OPN levels. Below the graphs are representative western blots for the respective treatment groups and time points. For western blots, CS = contralateral tibia saline control group, CA = contralateral tibia alcohol-exposed group, FS = fracture callus from saline control group, and FA = fracture callus from alcohol-exposed group. Bar graph are shown as mean ± SEM, *n* = 3–4/group for contralateral, and 8–9/group for fracture callus. *p < 0.05 by one-way ANOVA with Tukey’s post hoc test

**Fig. 6:**
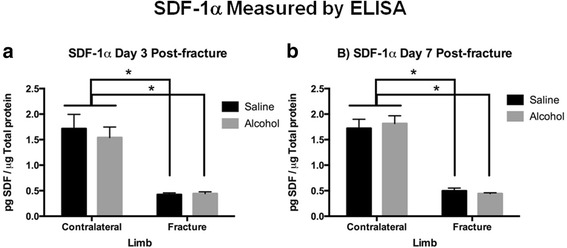
SDF-1α Protein Levels in Fracture Callus at **a** 3 days and **b** 7 days Post-Fracture. Bar graphs depict levels of SDF-1α in fracture callus or intact tibia specimens as pg SDF-1α per μg of total protein in the sample as measured by ELISA. Contralateral = intact non-fractured tibia, Fracture = callus from fractured tibia, Alcohol = episodic alcohol exposure, and Saline = control mice. Data shown as mean ± SEM, *n* = 3/group for contralateral, and 9/group for fracture callus. *p < 0.05 by one-way ANOVA with Tukey’s post hoc test

OPN protein expression was examined by western blot analysis in fracture callus samples from saline control and alcohol-exposed animals. The uninjured, contralateral saline control tibiae were used to scale OPN protein levels for semi-quantitative analysis. OPN was significantly decreased (*p* < 0.05) in fracture callus, regardless of treatment at day 3 post-fracture compared to the contralateral uninjured limbs (Fig. 5a). At day 7 post-fracture, OPN protein levels were significantly increased (*p* < 0.05) in the fracture callus of the saline control compared with contralateral uninjured limbs (Fig. 5b). This increase in fracture callus-associated OPN expression at day 7 post-injury was significantly blunted (*p* < 0.05) in alcohol-exposed mice. SDF-1α was assayed in callus samples by sandwich ELISA, and values were normalized to microgram of total protein. At both 3 and 7 days post-fracture SDF-1α expression was significantly decreased (*p* < 0.05) in fracture calluses compared to the contralateral uninjured limbs (Fig. 6a, b). There was no effect of alcohol exposure on SDF-1α expression in fracture callus tissue at post-injury days 3 or 7.

### Effects of alcohol on in vitro MSC migration and integrin β1 expression

**Fig. 7:**
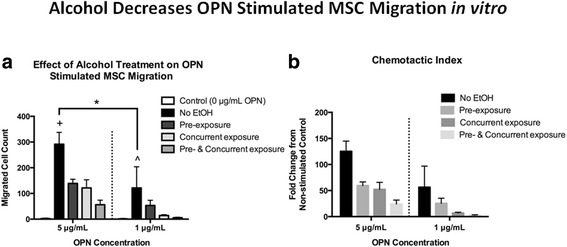
Effect of alcohol on in vitro MSC migration. Bar graphs show **a** migration of primary MSCs as a function of rOPN concentration and alcohol treatment and **b** chemotactic index of MSC migration data. Control = MSC migration in the absence of rOPN, no EtOH = rOPN stimulated MSC migration with no exposure to alcohol, pre-exposure = 24 h. pre-incubation of MSC in 50 mM EtOH, concurrent exposure = MSC migration assay performed in the presence of 50 mM EtOH in the rOPN well, pre- and concurrent exposure = combination of pre-exposure and concurrent exposure treatments. Data are shown as mean ± SEM, *n* = 4 experiments/group, each performed in triplicate. A two-way ANOVA with Holm-Sidak post hoc testing was performed on pre-defined comparisons of the no EtOH group versus every other bar at either the 5 or 1 μg/mL rOPN concentration and comparing no EtOH at the 5 and 1 μg/mL rOPN concentrations. *p < 0.05 comparing no EtOH at the 5 and 1 μg/mL rOPN concentrations. +*p* < 0.05 comparing no EtOH to all other bars in the 5 μg/mL rOPN group (to left of dashed line). All conditions showed statistically less MSC migration than the no EtOH group. ^*p* < 0.05 comparing no EtOH to all other bars in the 1 μg/mL rOPN group (to right of dashed line). All conditions except pre-exposure showed statistically less MSC migration than the no EtOH group. Each experiment was repeated at least three times utilizing unique primary MSC cultures

Prior research has shown that OPN acts as a chemokine to facilitate MSC migration via the integrin β1 receptor [32, 44]. We examined whether alcohol exposure would affect OPN-mediated MSC migration in vitro. First, we demonstrated that primary cultured mouse MSC migrated toward rOPN (5 or 1 μg/ml) in a dose-dependent manner, with negligible migration observed in cells not stimulated with rOPN (Fig. 7a). Primary MSC were then cultured in the presence of 50 mM ethanol for either 24 h prior to the assay (pre-exposure), during the assay (concurrent exposure), or both. In each exposure regimen, MSC demonstrated significantly less migration toward 5 μg/ml rOPN. (Fig. 7a). A trend toward decreased MSC migration was observed in cells exposed to 50 mM ethanol and stimulated with 1 μg/ml rOPN (Fig. 7a). The chemotactic index, expressed as fold change in MSC migration over unstimulated control MSC migration is shown in Fig. 7b.

**Fig. 8:**
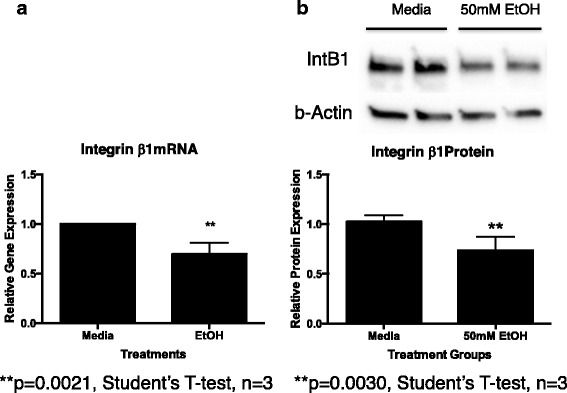
Effect of alcohol on primary cultured MSC integrin β1 mRNA and protein expression. Primary rat MSC were cultured in media alone or media plus 50 mM ethanol for 24 h. Cells were harvested and used for **a** mRNA or **b** total protein isolation as described. Int β1 mRNA levels were assessed by qRT-PCR as described. Int β1 protein levels were assessed by western blot analysis as described. **a** mRNA: media vs EtOH *p* = 0.0021. **b** Protein: media vs EtOH *p* = 0.0030. Each experiment was repeated at least three times utilizing unique primary MSC cultures. *p < 0.05 by Student’s *t* test

In an attempt to determine why ethanol exposure caused a decreased migration of primary MSC toward rOPN, we examined integrin β1 expression in primary MSC cultured in the presence of 50 mM ethanol for 24 h. We found that both mRNA (Fig. 8a) and protein levels (Fig. 8b) for the integrin β1 receptor were significantly decreased in MSC exposed to 50 mM ethanol in vitro (*p* = 0.002 and 0.003, respectively). In contrast, ethanol exposure did not significantly alter the expression of CD44 (another OPN receptor) in cultured MSC (data not shown).

## Discussion

In this study, we examined the effects of episodic alcohol exposure on the early stages of fracture healing in a model system of mouse tibia fracture. We show evidence that the accumulation of granulation tissue and mineralization of the external cartilaginous callus forming at the fracture site are negatively affected by alcohol exposure. We also demonstrated decreased fracture callus biomechanical stiffness at day 7 post-injury. Because MSC localization to the fracture site is critical to external callus formation, we examined the effects of alcohol on chemokine expression in the early callus and showed significantly decreased levels of OPN in callus from alcohol-exposed mice at 7 days post-injury. Finally, we demonstrated that alcohol exposure decreases MSC integrin β1 receptor expression and blunts osteopontin-induced MSC migration in vitro. Taken together, these results suggest that alcohol-related inhibition of fracture callus formation may be related in part to its perturbation of OPN-mediated MSC localization to, or activity at, the fracture site. Our observations that alcohol exposure decreases OPN expression during early bone fracture healing and that exposure of cultured MSC to alcohol alters integrin β1 receptor levels and inhibits the migration of stem cells toward rOPN have not been previously reported. Although our episodic alcohol regimen likely causes a delay in the fracture repair rather than a non-healing fracture, the effects of alcohol observed on early fracture repair may have important repercussions whether the final outcome is delayed healing or non-union [45].

The effects of alcohol we observed on fracture callus histology, microstructure, and biomechanical parameters provide evidence that alcohol negatively affects the early fracture healing process. Our histological data demonstrates a qualitative effect of pre-and post-injury episodic alcohol exposure on early accumulation of granulation tissue at the fracture site. The observations reported here on the effects of pre- and post-fracture alcohol exposure are in line with our previously published data on pre-injury alcohol exposure and callus formation [18, 19, 43]. Further, while total volume of the callus did not differ between groups (as measured by μCT), the percentage of newly mineralized tissue was significantly lower in alcohol-exposed animals than in saline controls. We have previously shown decreased new bone volume at the callus in response to episodic alcohol exposure at 14 days post-injury [20]. Our current data demonstrates that this effect on callus mineralization occurs as early as 7 days. Fracture callus from alcohol-treated mice was less stiff in 4-point bending than samples from corresponding saline-treated mice. Control callus stiffness values obtained in our study were similar to those at 7 days post fracture obtained by Hiltunen [46]. Thus, callus from saline control mice is of better quality than from episodic alcohol-exposed mice, reflected by the differences in cartilaginous callus observed histologically [43] and newly mineralized tissue measured via μCT. Stiffness of a callus is affected by tissue amount, composition, geometry, or a combination of factors. The decreased stiffness of calluses from alcohol-treated mice likely relates to a decreased percentage of newly mineralized tissue as it is not due to changes in callus volume or distribution of the mass, as neither TV nor *I*_pol_ were affected by alcohol exposure.

We have previously demonstrated attenuation of Canonical Wnt signaling activity at the fracture site in alcohol-treated mice [18, 19], suggesting that alcohol may disrupt signaling through a cellular pathway important for MSC differentiation [47] and subsequent fracture healing [32]. The data presented here suggest that alcohol also affects cellular signaling important for MSC localization to the injury site. Both OPN and SDF-1α are chemokines expressed at the fracture site which have previously been shown to be involved in MSC migration [30–32, 44]. At 3 days post-injury, callus-specific expression of OPN and SDF-1α were not affected by alcohol exposure in our model. However, alcohol exposure decreased callus-associated OPN levels 7 days post-injury compared to the normal increase seen in saline controls. This observation suggests that an alcohol-specific perturbation of OPN-mediated chemokine signaling could underlie, at least in part, the deficits in cartilaginous callus formation we have previously observed in alcohol-exposed animals [43] by affecting either MSC availability or activity at the site of injury. While these specific chemokine levels at the fracture site appear not to be perturbed by alcohol at 3 days post-injury, we cannot rule out that other early chemokine-related signaling in MSC may be affected by alcohol during early fracture repair.

## Conclusion

A recent clinical study showed that serum OPN levels were increased at 7 days post-injury in patients sustaining a long bone fracture [48]. OPN knockout mice show decreased callus volume, reduced callus biomechanical properties and increased callus mineralization as compared to wild-type mice [49]. This report suggests that OPN expression at the fracture site may be related to callus volume and biomechanical strength of the callus, which agrees with our data. The exact role of OPN in bone mineralization is not clear [50], and, given its multiplicity of known functions, it could have different functions at different times post-fracture. Alcohol has other known effects on signaling activity at the fracture site [18, 19, 51], which complicates any direct comparisons between our study and those utilizing OPN knockout animals. OPN has other important roles in bone including the modulation of hydroxyapatite formation during bone mineralization [50], so any alcohol-related attenuation of OPN activity could have other effects on fracture healing unrelated to its chemokine-related activity. Although we did not observe alcohol-specific effects on SDF-1α levels in the fracture callus in our study, a prior investigation demonstrated that SDF-1α directs MSC migration after fracture [30]. This study used a bone-grafting model of bone repair and measured the time course of SDF-1α RNA expression by qPCR. While we did not show either increases in SDF-1α protein levels following fracture or alcohol exposure-related effects on SDF-1α levels in the callus, we cannot rule out that SDF-1α-dependent MSC migration to the fracture site could be occurring in our model.

The biological relevance of MSC migration to the fracture site during fracture repair is not understood [52]. Osteopontin has several potential biologic functions during fracture healing, including participation in angiogenesis [48, 49], stem cell recruitment [31, 32, 44], stem cell differentiation [53, 54], and mineralization [50]. Our hypothesis that OPN-stimulated MSC migration may be a target of alcohol exposure was examined here in vitro, showing *a* dose-dependent migration of MSC toward OPN, and that exposure of MSC to alcohol blunts this response. To our knowledge, there are no other reports to date on the effects of alcohol exposure on MSC migration. Experiments are currently in progress to examine the effects of alcohol on the migration potential of patient-derived bone marrow MSC. Previous studies have shown that stem cells obtained from patients with alcohol-induced osteonecrosis of the femoral head show reduced ability to differentiate toward an osteogenic lineage compared with MSC obtained from patients with femoral neck fractures (55), suggesting that alcohol abuse may cause global changes in MSC function, leading to skeletal diseases such as osteonecrosis and fracture non-union.

It has previously been shown that MSC migrate toward OPN via a CD44-mediated pathway stimulated by hypoxic osteocytes [31]. Of note, during the very early stages of fracture healing, damage to local vasculature can render the fracture site hypoxic relative to surrounding tissues [55]. Other reports suggest that OPN-mediated MSC migration occurs via interaction with the integrin β1 receptor [32, 44]. Our data showed that OPN stimulates primary MSC migration in a dose-dependent fashion in vitro, and that alcohol inhibited this migration. We demonstrated that alcohol treatment of primary MSC significantly decreased both mRNA and protein levels of integrin β1. This data may partially explain the mechanism underlying the ethanol-related decrease in OPN-mediated MSC migration shown in the in vitro system. Coupled with data showing decreased OPN expression in callus tissue from alcohol-treated mice, the data suggests that OPN-related signaling is targeted by alcohol exposure during the early repair period.

Limitations to the current study include effects of alcohol intoxication on post fracture animal activity and the fracture fixation technique. The administration of alcohol at an intoxicating level could modulate ambulatory profiles of mice and differences in post-injury activity could alter biomechanical loading at the fracture site and subsequently affect fracture repair [56]. While we did not monitor rodent activity, alcohol administration was performed at the beginning of the light cycle, giving animals several hours to metabolize alcohol prior to the dark cycle and the period of greatest activity for rodents. With respect to fixation, the tibial intramedullary pin technique [18–20] allows the injured bone ends to remain in close proximity during healing and provides reasonable stability. Appliances for the rigid fixation of rodent fractures are available [57] and would eliminate variables associated with fixation, but rigid fixation results in cortical bridging of the fracture via intramembranous bone formation without appreciable external callus formation. As we believe that inhibition of external callus formation may be the primary defect in alcohol-exposed rodents, the use of a rigid fixation device would not be appropriate for the current studies. Mice were eliminated from the study if any evidence of pin migration was found during fracture callus collection, which could result in inadequate fixation, ensuring that all specimens utilized were appropriately stabilized.

From a clinical perspective, the current study is limited primarily by the fact that it is a laboratory animal study. However, our results could impact clinicians involved in the treatment of delayed fracture union/non-union, because the study identifies a potential novel mechanism underlying alcohol-related delayed fracture healing (fracture-associated chemokine expression and MSC migration), which could be amenable to targeted stand-alone therapies or as an adjunct to surgical procedures for non-union. This study also provides information regarding the role of alcohol abuse as a modifiable risk factor in fracture healing, and data from this investigation may eventually lead to targeted pharmacologic or cell-based therapies that restore fracture healing in patients suffering from alcohol abuse disorders without the need for surgery.

## Abbreviations

BAL: Blood alcohol level
BV: Bone volume
BW: Body weight
EtOH: Ethanol
IP: Intraperitoneal
*I*_pol_: Polar moment of inertia
MSC: Mesenchymal stem cells
OPN: Osteopontin
SDF1-α: Stromal cell-derived factor-1
TV: Total volume
